# Facilitating autonomous, confident and satisfying choices: a mixed-method study of women’s choice-making in prenatal screening for common aneuploidies

**DOI:** 10.1186/s12884-018-1752-y

**Published:** 2018-05-02

**Authors:** An Chen, Henni Tenhunen, Paulus Torkki, Antti Peltokorpi, Seppo Heinonen, Paul Lillrank, Vedran Stefanovic

**Affiliations:** 1 Institute of Healthcare Engineering, Management and Architecture (HEMA), Department of Industrial Engineering and Management, Aalto University, Maarintie 8, P.O. Box 15500, 00076 Espoo, Finland; 2 Department of Civil Engineering, Aalto University, Rakentajanaukio 4, 02150 Espoo, Finland; 3 Department of Obstetrics and Gynaecology, Helsinki University Hospital and University of Helsinki, Haartmaninkatu 2, 00290 Helsinki, Finland; 4 Department of Public Health, Faculty of Medicine, Helsinki University, Biomedicum 1, 00290 Helsinki, Finland

**Keywords:** Women’s choice, Choice-making experience, Choice satisfaction, Prenatal screening

## Abstract

**Background:**

Population-based prenatal screening has become a common and widely available obstetrical practice in majority of developed countries. Under the patient autonomy principle, women should understand the screening options, be able to take their personal preferences and situations into account, and be encouraged to make autonomous and intentional decisions. The majority of the current research focuses on the prenatal screening uptake rate, women’s choice on screening tests, and the influential factors. However, little attention has been paid to women’s choice-making processes and experiences in prenatal screening and their influences on choice satisfaction. Understanding women’s choice-making processes and experiences in pregnancy and childbirth is the prerequisite for designing women-centered choice aids and delivering women-centered maternity care. This paper presents a pilot study that aims to investigate women’s experiences when they make choices for screening tests, quantify the choice-making experience, and identify the experiential factors that affect women’s satisfaction on choices they made.

**Method:**

We conducted a mixed-method research at Helsinki and Uusimaa Hospital District (HUS) in Finland. First, the women’s choice-making experiences were explored by semi-structured interviews. We interviewed 28 women who participated in prenatal screening. The interview data was processed by thematic analysis. Then, a cross-sectional self-completion survey was designed and implemented, assessing women’s experiences in choice-making and identifying the experiential factors that influence choice satisfaction. Of 940 distributed questionnaires, 185 responses were received. Multivariable linear regression analysis was used to detect the effects of the variables.

**Results:**

We developed a set of measurements for women’s choice-making experiences in prenatal screening with seven variables: activeness, informedness, confidence, social pressure, difficulty, positive emotion and negative emotion. Regression revealed that activeness in choice-making (*β* = 0.176; *p* = 0.023), confidence in choice-making (*β* = 0.388; *p* < 0.001), perceived social pressure (*β* = − 0.306; *p* < 0.001) and perceived difficulty (*β* = − 0.274; *p* < 0.001) significantly influenced women’s choice satisfaction in prenatal screening.

**Conclusions:**

This study explores the experiential dimension of women’s choice-making in prenatal screening. Our result will be useful for service providers to design women-centered choice environment. Women’s willingness and capabilities of making active choices, their preferences, and social reliance should be well considered in order to facilitate autonomous, confident and satisfying choices.

**Electronic supplementary material:**

The online version of this article (10.1186/s12884-018-1752-y) contains supplementary material, which is available to authorized users.

## Background

As a critical component of the delivery of high-quality and evidence-based prenatal care [1], population-based prenatal screening has become a common and widely available obstetrical practice in majority of developed countries [2–5]. It provides women and families with a risk figure that estimates the chances of having a baby with chromosomal abnormalities and allows them to make decisions and preparations in a timely manner [1, 4, 6]. There are different screening options. For example, all pregnant women in Finland are entitled by law for the voluntary first trimester combined screening (FTS) at 9–13^+ 6^ weeks of pregnancy and second trimester genetic sonogram performed at 18^+ 0^–22^+ 0^ weeks of pregnancy. For those who have missed the FTS a second trimester maternal serum screening (STS) is offered at 15–16^+ 6^ gestational weeks. The FTS takes into account the mother’s age and weight, fetal crown-rump length and nuchal translucency thickness measured by ultrasound as well as results from maternal serum biomarkers [*free β-human chorionic gonadotropin (β-hCG), pregnancy-associated plasma protein-A (PAPP-A*)]. STS is based on maternal serum β-hCG and alpha fetoprotein (AFP) adjusted for the gestational age determined by ultrasound. This method is suitable those who have missed FTS for any reason. Cell-free fetal DNA screening is offered as the second-tier screening in public sector for those who are screen-positive at FTS or STS.

Because the positive result at prenatal screening for fetal aneuploidies leads frequently to the invasive diagnostic procedures and possible termination of affected pregnancies [7], autonomous choice and decision, i.e. informing women about all existing tests and allowing women to make choices and take actions that are based on their personal willingness, beliefs, values and other considerations without any coercion, have been highly recognized [8, 9]. The assumption of this autonomy principle is that women should be able to understand the screening programs, be clear about their situations, and have the capability to make autonomous and intentional decisions [8]. This is not possible to all women, due to plethora of reasons. It has become a high priority to understand women’s choice and choice-making, for the purpose of building appropriate choice platforms, assisting women in making choices, and delivering patient-centered care [10].

A growing body of research focuses on the uptake rate of prenatal screening, women’s choice on screening tests, and the influential factors of the choice. Bakker et al. [11] evaluated the factors affecting the low uptake of FTS in the Netherlands and reported that the main reasons for the low uptake were the relatively positive attitude towards Down syndrome and a negative attitude towards pregnancy termination. With the same context, Crombag et al. [12] found that advanced maternal age and age related risk perception were strongly affecting women’s participation in prenatal screening. Košec et al. [13] conducted a cross-sectional study on a prospective basis in different districts of Croatia, assessing pregnant women’s awareness, motives and preferences about the first and the second trimester screening in early pregnancy before they chose any of the recommended tests. They found that education level and previous information were significant variables predictive for the choice of the tests. Informed choice, a choice that is based on relevant knowledge, consistent with decision-maker’s values and behaviorally implemented [14], has been regarded as a central feature of screening and testing [15]. The research on informed choice in prenatal screening has become very popular. For example, Marteau et al. [14] developed a multi-dimensional measure of informed choice for women undergoing prenatal screening; Potter et al. [16] employed the measurement and qualitatively evaluated women’s choice in their case; Schoonen et al. [6] analyzed the content of decision-relevant knowledge and produced a knowledge structure that can facilitate the informed choice in prenatal screening.

Patient-centered health services cannot be achieved without understanding patient experience and satisfaction [17]. The experiences of pregnancy and childbirth can have immediate as well as long-term effects on women’s life, well-being and health [18, 19]. However, only few studies have estimated women’s choice-making processes in prenatal screening [20] or explored the choice-making experiences in order to design women-centered choice aids and deliver women-centered maternity care. From a service perspective, experience can be understood as a product of sense-making, which is subjective, interpretive, and individually, contextually and socially-constructed [21–23]. In health service context, patient as a social actor with subjectivity perceives the phenomena, makes sense of events and constructs the experience. Larkin et al. [19] study the concept of experience in the context of pregnancy and childbirth and broadly define experience as “an individual life event, incorporating interrelated subjective psychological and physiological processes, influenced by social, environmental, organizational and policy contexts.” However, the current research on women’s choice-making processes and experiences has a narrow set of focuses on choice conflict between wishes, preferences and ethical views [9], anxiety and depression affecting choice-making [24], the difficulty of making choices [25] or information needs [26]. There is no comprehensive study deeply exploring women’s choice-making experiences in prenatal screening. The main objective of the study is to explore women’s experiences when they make choices for screening tests, quantify the choice-making experience, and identify the experiential factors that affect women’s satisfaction on choices they made (choice satisfaction). We hope this study can advance the discussions on how to implement screening program, how to counsel pregnant women and how to assist women in making informed and autonomous choices. The experience of this study could also be shared for other medical contexts (e.g. cancer screening) to explore the experiential dimension of patient choice-making.

## Methods

### Empirical context

We conduct a pilot study to investigate women’s choice-making experiences in population-based prenatal screening within the public maternal service system at the Department of Obstetrics and Gynecology, Helsinki and Uusimaa Hospital District (HUS) Finland, where aforementioned non-invasive screening tests (FTS and STS) are offered free of charge to all women. Finnish community-based maternity care unit (Neuvola), a public sector activity led by general practitioners and providing free maternal care service to all pregnant women, is responsible for informing pregnant women about prenatal screening and testing program and helps to arrange the tests. Every pregnant woman receives a booklet (Additional file 1) of prenatal screening and testing from the nearest maternity care unit. This study was approved by the Ethics Committee for Gynecology and Obstetrics, Pediatrics and Psychiatry, Helsinki University Hospital (permission number: 220/13/03/03/2015).

### Study design

We applied a mixed-method approach using qualitative data and quantitative data in one study. This could lead to greater validity and rigor and provide a better understanding of the research problem than either research approach alone [27]. Many researchers have agreed that the mixed methods approach can be particularly useful in healthcare research that requires a broader range of perspectives to view the complexity in this field [28–30]. We followed the sequential exploratory design [31], in which qualitative data are collected and analyzed first, followed by quantitative study [32, 33]. This design is useful for exploring the unknown variables, developing new instruments based on an in-depth qualitative analysis, and confirming or generalizing qualitative findings by quantitative study. This is suitable to our case, where the complex phenomenon of women’s choice-making experiences needs structuralization and operationalization. We explore how women experience the choice-making process in prenatal screening (qualitative research question), and identify what are the experiential factors significantly influencing women’s choice satisfaction (quantitative research question).

### Qualitative study

The details of women’s choice-making experiences were explored by semi-structured interviews. Storytelling or narrative inquiry as a way of investigating the subjective experience and delving into individual’s “lifeworld” [34–36] produced remarkable contributions for the qualitative study. The inquired modes of experience were quite broad, including individual’s feeling, seeing, hearing, reflecting, expecting, acting, and all other kinds of emotional, mental and physical activities. Interviews, that were conducted after the women had gone through the screening, started with open-ended questions about how the woman made a choice between different screening tests. The concrete questions were developed from Theory of Planned Behavior (TPB) [37, 38], which helps to understand the choice-making process from women’s perspective with three main constructs: behavioral beliefs, subjective norms and perceived behavioral control. “Behavioral beliefs” refers to personal evaluation of a behavior (e.g. choosing the FTS) and by this we explored women’s reflections on the advantages and disadvantages of the choice consequences (e.g. the consequences of choosing the FTS). “Subjective norms” refers to social influence and by this we investigated how women’s choices were socially affected by other people. “Perceived behavioral control” refers to the resources and opportunities available to person to make a certain behavior (choice) possible and in this study it helped to evaluate the ease and difficulty of making the choice. Midwives from the Screening Unit and Fetomaternal Medical Center (FMC) in Helsinki helped to recruit the interviewees who participated in prenatal screening by presenting the information letter explaining our study to women and inviting them to participate in the study on a voluntary basis. All the informants voluntarily participating in the interviews signed a written informed consent. Qualitative data collection were collected in a period of December 2015–May 2016. Interviews were conducted in Finnish (author HT conducted) or English (AC conducted). Interviews were tape recorded, transcribed in English and imported into Atlas.ti qualitative data analysis software. The data were processed by thematic analysis. Author AC and HT independently coded the data, identifying the items that could delineate women’s choice-making processes and experiences. Then the two authors discussed, reached an agreement on the items and categorized them into the themes. We enhanced the validity of our findings by organizing discussions in the research team, consulting experts from the Screening Unit, and getting feedbacks from professionals. In total, we interviewed 28 women.

### Quantitative study

Following the guidelines of scale development provided by DeVellis [39], we developed a framework of measurements for women’s choice-making experiences in prenatal screening and designed a cross-sectional self-completion survey on a retrospective basis to assess women’s experiences in choice-making processes. The generation of measurements for choice-making experience was based on the themes of choice-making process and experience uncovered by the qualitative study as well as some current validated measurements. In the questionnaire, we also measure women’s satisfaction on the choices they made as the primary outcome measurement. Items for measurements were made in a statement format with 5-point Likert scale from “strongly disagree” to “strongly agree”. We asked participants to provide demographics information, including maternal age, education level, marital status, occupation, gravidity and previous experiences of fetal aneuploidy. The questionnaire was critically reviewed and approved by the expert in fetomaternal medicine (VS) who was responsible for the organization of prenatal screening at HUS at the time of commencing this study. In order to test and improve the quality of the measurements, we invited three women to fill in the questionnaire and asked them to report their understandings, confusions and difficulties when responding to each item. Questionnaire was made in English and then translated into Finnish and Swedish. Translation service agency, researchers and medical professionals co-worked in translation and back-translation. Our questionnaires were distributed to the women who participated in prenatal screening. Midwives from delivery hospitals, in which certified midwives perform screenings for pregnant women, helped to disseminate the anonymous questionnaires in the Screening Unit where screening tests and ultrasound are performed. The survey lasted 10 months, from June 2016 to March 2017. Factor analysis was employed to further validate the measurements. Factor analysis empirically determined the number of dimensions/constructs underlying a set of items and refined the inclusion of items for each dimension [39]. Each variable of choice-making experience was produced by summating its measurement items and assessed by one-sample t test, testing whether the variable mean was equal to a neutral value. Multivariable linear regression analysis was used to detect the effects of these variables on choice satisfaction. Quantitative data was managed and analyzed by STATA 13. A *p*-value of < 0.05 was used to establish statistical significance.

## Results

### Qualitative study results

We interviewed 28 women who participated in prenatal screening, of which 22 (78.6%) chose FTS and 6 (21,4%) participated in STS. Table 1 displays the six dimensions of women’s choice-making processes and experiences in prenatal screening: activeness (attitude and behavior), informedness (information needs and sources), preferences/considerations, social influence (perceived social support and pressure), difficulty (difficulty in making choice) and emotional status. For each item under these dimensions, we counted the number of women mentioning it to evaluate its importance, and pictured the choice-making differences between the two groups of women participating in different screening tests. Sample quotations are shown in Table 1.

**Table 1:** Six dimensions of women’s choice-making processes and experiences in prenatal screening

Choice-making dimensions	First trimester combined screening (22)	Second trimester screening (6)
Active choice	didn’t make active choices (16) *“I didn’t choose the tests actively. I didn’t know much about the second trimester serum screening and I just participated in what was recommended”* made active choices (6) *“I made an active choice. It was my decision to participated in the first trimester combined screening, which is better than the other test”*	didn’t make active choices (5) *“I didn’t choose the tests actively. The participation in screening is nothing, you just go and do what they (public health nurses) arrange for you.”* made active choices (1) *“I made an active choice. I consulted about the different options and I preferred the first trimester combined screening. But since I was traveling during the screening week, so I had to do the second trimester serum screening.*”
Informedness	Information needs: the possible results of the tests (16); procedures of different programs (16); different options (10); the features, e.g. accuracy, risk and timing of the tests (7); advantages/disadvantage of tests (5); mechanisms and theories of tests (1) *“I got the main information from Neuvola, including the practical information about the screening and its possible results, what it includes in the program and what the ultrasound means. This information is very important.”* Information sources: Booklet in maternity care unit (16); Public health nurse of maternity care unit (10); Previous experience (8); Online information (4); Family members (1); Friends (1); *“Information was given by Neuvola for sure, with the information about what it is and what is the expected outcome. I didn’t seek for information from other sources by myself.”*	Information needs: different options (6); the possible results of the tests (5); procedures of different programs (4); the features, e.g. accuracy, risk and timing of the tests (3); advantages/disadvantage of tests (3); mechanisms and theories of tests (0) *“I could not take the first trimester screening. The midwife told me about the second trimester screening. The timing and schedule information were very important in my case.”* Information sources: Booklet in maternity care unit (6); Public health nurse of maternity care unit (6); Online information (2); Previous experience (2); Family members (1); Friends (0); *“The nurse told me about the second trimester screening. I did check the information about screening from internet after that (after I decided to do it). I didn’t ask any friends.”*
Main preferences/considerations	Reliability/Accuracy (19): the result is more reliable; certainty is higher; more information is provided. *“Combined screening can give more information than just doing blood test”* Easiness (18): the procedure is simple *“It is quite easy. Some problems can be directly detected when I take the ultrasound.”* Recommendation (17): the test is recommended by trusted persons. *“The Neuvola* nurse *recommended the ultrasound to check if everything was ok.”* Timing (10): the test can be organized in good time or suitable to the client’s schedule. *“The nurse of Neuvola helped to book the time of blood testing and ultrasound, which is suitable to my schedule.”* Rapidness (8): to get the test and result quickly with a short waiting time *“Combined screening can give earlier information and the ultrasound can detect the problems very quickly, so that we can make preparations or take further steps earlier if there is any problem.”*	Timing (6): the test can be organized in good time or suitable to the client’s schedule. *“I have scheduled a trip during that week, so I could not participate in the first trimester screening. Only the second trimester works for me. I missed the first trimester screening and I had little time left to do the screening”* Reliability/Accuracy (3): the result is more reliable; certainty is higher; with more information. *“I preferred the first trimester screening at the beginning because it can offer more reliable and early result. But I missed it because of the travelling. So I have to rely on the second trimester screening”* Recommendation (2): the test is recommended by trusted persons. *“The Neuvola nurse suggested that I could do the second trimester screening based on my situation.”* Easiness (2): the procedure is simple *“Just taking blood sample is quite easy”* Access (1): the option is available and offered freely *“It is not free to do the screening abroad. We participated in the second trimester screening when we came back to Finland.”*
Social influence	Perceived social support (19) *“I agreed with the midwife’s arrangement. I also talked with my husband and it was a common decision. My husband supported me”* Perceived social pressure (8) *“Some of my friends experienced difficult pregnancies, so I want to participate in the screening and get the information earlier.”* Social influence from who: Medical staff (18); Husband/partner (4); Friends (1); Family (1); *“The Neuvola nurse discussed about prenatal screening and testing with me and provided the information to me. She recommended the first trimester screening.”*	Perceived social support (3) *“The Neuvola nurse gave me suggestion and helped to arrange everything”* Perceived social pressure (1) *“Everyone participates in screening. I felt I have to do something for the baby. I missed the first trimester combined screening, and I felt upset and disappointed.”* Social influence from who: Medical staff (3); Husband/partner (1); Family (1); Friends (0); “*My husband was with me in the Neuvola visit and we made the decision together*.”
Difficulty in making choice	it was not difficult to make a choice (22) *“I didn’t have any difficulties. It is a kind of routine. I just followed what the nurse recommended”*	it was not difficult to make a choice (4) *“I was quite clear about choice and just followed the suggestion (given by the Neuvola nurse)”* it was difficult to make a choice (2) *“It was not easy to make decision, because of the information insufficiency. I was traveling in the beginning of the pregnancy. I thought I could do the screening abroad but it was difficult to organize. I didn’t get clear instruction from Neuvola for my situation.”*
Emotional status	Neutral (20) *“It is just something straightforward and obvious to do. I didn’t feel there is something wrong.”* Positive (3) *“I felt content that I can choose the better one”* Negative (1) *“I was worried about the possible risks and bad results.”*	Positive (1) *“I missed the first trimester screening, but I felt relieved the other test was still possible.”* Negative (3) *“I was annoyed that I missed the combination screening.”*

#### Active choice

Our participants had a common understanding on active choice, referring to three major constructs: information collection, autonomy and consciousness. Information collection (mentioned by 21 women) means that the woman reads the information, knows all the options and actively searches information from different sources. Autonomy (mentioned by 18 women) means that the woman can freely decide everything by herself based on individual willingness and that she is not forced to do something or to make the decision. Consciousness (mentioned by 6 women) implies that the woman thinks about the options, questions the options and consciously decides between the alternatives. In our study, most of the women reported that they did not actively choose the screening tests, because they were not aware of the STS, they thought it was the default choice to participate in the FTS, or they felt that there was little risk involved in the process.

#### Informedness

A key finding from our analysis was that, women had different information needs for choosing screening tests. Most of them were aware of the procedures and possible results of the tests. More than half of the women expressed they would have wanted to know that there was a later screening possibility. Some of them considered the features (e.g. accuracy, risk and timing) or the advantages/disadvantages of the tests. Few of them were concerned about the mechanisms and theories of the tests. Maternity care unit booklet and public health nurse were the two main information sources. Some women had previous experience in screening. Few women sought additional information online or other sources.

#### Preferences/considerations

In the interviews, we asked women what mattered to them for screening and explored their main preferences and considerations. In the table, we listed the prominent preferences/considerations of each group. For most women participating in the FTS, reliability/accuracy was the most important issue (mentioned by 19 women), referring to the reliable test results, high certainty and extensive information. The second most important factor was easiness (mentioned by 18 women), referring to the simplicity of the test performance. Recommendation (mentioned by 17 women) given by the public health nurses of maternity care unit was the third important issue. Other prominent preferences included timing (mentioned by 10 women), which means the test can be organized in good time or suitable to the client’s schedule, and rapidness (mentioned by 8 women), which means to get the test and result quickly with a short waiting time. For women participating in the STS, timing (mentioned by 6 women) was the most important factor. Test accuracy (mentioned by 3 women) and recommendation (mentioned by 2 women) were also considered.

#### Social influence

Our participants recalled whether their choice-making was influenced by other people (including health professionals, e.g. midwives, as well as other people in the society, e.g. husband/partner), and whether their choice got any support or pressure. Most of women who participated in the FTS followed the suggestions from maternity care units without much doubts or discussing seriously with others. Some of them made the decision with their husband or partner. Generally, women did not feel much pressure from others and they found it easy to follow the arrangement suggested by maternity care units or do as other women do. One woman mentioned that she felt some pressure, because her friends experienced difficult pregnancies. Among the women participating in the STS, three mentioned that the public health nurses of maternity care unit gave advice and helped to arrange the tests. Only one woman said that she felt some pressure of making the choice, thinking about other people and their choices.

#### Difficulty

All women participating in the FTS reported that it was not difficult to make a choice. Some women said they chose combined screening as the first and best option without any hesitation. In contrast, not all women participating in the STS felt it was easy to make choices. Two women complained that they did not get enough information or instructions for their special situation, traveling aboard at the beginning of the pregnancy.

#### Emotional status

The interviewed women also reflected on their emotional status when making choices. The majority of women who participated in the FTS described that they felt quite neutral when making choice on screening tests. Many women said it was something very straightforward and obvious to do and nothing seemed to be going wrong. Some women reported positive feelings, e.g. being content for choosing the better screening option. One woman was worried about the possible risks and bad results. Some women who missed the FTS felt upset, disappointed or anxious. One woman said that she felt relieved when she discovered there was a backup option for the screening.

### Quantitative study results

Starting from June 2016, 940 questionnaires were distributed, 185 were returned by March 2017 with an overall response rate of 19.7% and 183 were valid. In our sample, 163 (89.1%) women participated in the FTS, 3 (1.6%) participated in the STS and 17 (10.4%) did not know which screening test they participated in. The demographic characteristics of the sample are shown in Table 2.

**Table 2:** Sample characteristics of the survey

Demographic characteristics	Sample size = 183
Mean age (years) (SD)	32 (4.46)
Education (*n*) (%)	
1. Basic education or less	1 (0.55)
2. Secondary education or vocational qualifications	33 (18.03)
3. Bachelor level	54 (29.51)
4. Master level	82 (44.81)
5. Licentiate or doctor’s degree	13 (7.10)
Marital status (*n*) (%)	
1. Single	10 (5.46)
2. Married	101 (55.19)
3. Co-habitation	71 (38.80)
4. Divorced	1 (0.55)
Occupation (*n*) (%)	
1. Student	13 (7.10)
2. Labor worker	79 (43.17)
3. Lower white-collar worker	18 (9.84)
4. Upper white-collar worker	53 (28.96)
5. Entrepreneur	6 (3.28)
6. Unemployed	14 (7.65)
7. Pensioner	0 (0)
Gravidity, including current pregnancy (times) (SD)	1.99 (1.20)
Chromosomal abnormality in previous pregnancy (*n*) (%)	
1. with chromosomal abnormality in previous pregnancy	2 (1.09)
2. no chromosomal abnormality in previous pregnancy	181 (98.91)

The design of the choice-making experience measurements was based on the results of the qualitative study, group discussions and validated measurements including Decisional Conflict Scale [40, 41] (Additional file 2) and six-item short-form of the state scale of the Spielberger State-Trait Anxiety Inventory [42] (Additional file 3). Additional file 4 summarizes the initially developed constructs (7 constructs for choice-making experience and one for choice satisfaction) with the meanings, measurement items and score ranges. The constructs created by factor analysis (Additional file 5) for all the items measuring the choice-making experience were similar with the original conceptual ones, which means that the original categories have a certain level of validity to account for covariation among the items. One adjustment based on factor analysis was that the item “someone else made the choices for me” that was supposed to measure activeness of choice-making grouped with the measurement of social pressure. The second adjustment was that the items that were planned to measure preference consistency and the items that were planned to measure choice support grouped together as one factor, which was named with “choice-making confidence”. The third one was that the items reflecting positive emotional status and those reflecting negative emotional status did not belong to one emotion category, and they were divided into two factors. Thus, based on the factor analysis, a more structured and validated set of instruments measuring choice-making experience (Additional file 6) were generated, which included “active choice”, “informedness”, “confidence”, “social pressure”, “difficulty”, “positive emotion” and “negative emotion”.

Table 3 quantitatively outlines women’s choice-making experiences in prenatal screening. In general, the survey respondents did not have negative experiences when making choices for screening tests: they were well informed (mean: 22.41; score range: 7–35); they felt confident (mean: 16.83; score range: 5–25); they did not perceive social pressure (mean: 2.94; score range: 2–10); the perceived difficulty of making choices was very low (mean: 5.50; score range: 3–15); they had positive emotion status when making choices (mean: 9.93; score range: 3–15) and the anxiety level was low (mean: 17.62; score range: 9–45); but they did not actively make choices in prenatal screening (mean: 8.66; score range: 4–20). The choice satisfaction with the mean of 8.86 (score range: 2–10) was high.

**Table 3:** Exploring the variables of women’s choice-making experiences in prenatal screening

Variables	Score range	Neutral score	Mean score (SD)	*p* value of t test (mean score vs. neutral score)	Reliability (Cronbach’s alpha)
Activeness in choice-making	4–20	12	8.66 (3.78)	< 0.001	0.8022
Informedness	7–35	21	22.41 (7.67)	0.012	0.9412
Confidence	5–25	15	16.83 (5.24)	< 0.001	0.8778
Social pressure	2–10	6	2.94 (1.71)	< 0.001	0.6427
Difficulty	3–15	9	5.50 (2.60)	< 0.001	0.8489
Negative emotion	9–45	27	17.62 (7.62)	< 0.001	0.9277
Positive emotion	3–15	9	9.93 (3.03)	< 0.001	0.8681
Choice satisfaction	2–10	6	8.82 (1.58)	< 0.001	0.6673

Table 4 presents the result of the multiple linear regression identifying the experiential factors that significantly influence women’s choice satisfaction in prenatal screening. Demographic factors and screening results (normal, abnormal or do not know yet) at the point of filling in the questionnaire were input as control variables in the regression model. Regression revealed that education (β = − 0.136; *p* = 0.047), gravidity (β = − 0.182; *p* = 0.008), activeness in choice-making (*p* = 0.023), confidence (β = 0.388; *p* < 0.001), perceived social pressure (β = − 0.306; p < 0.001) and perceived difficulty (β = − 0.274; p < 0.001) significantly influenced women’s choice satisfaction in prenatal screening. Women with higher levels of education were more likely to have lower choice satisfaction. Women had lower choice satisfaction as the pregnancy times increased. If women made more active choices, choice satisfaction would increase. While choice-making confidence had strong positive effects on choice satisfaction, social pressure and perceived difficulty were negatively related to choice satisfaction.

**Table 4:** Multiple linear regression with choice satisfaction as the dependent variable

						95.0% CI for unstandardized coefficients	
Variables	Unstandardized coefficients	SE	Standardized coefficients	t	*p* value	Lower bound	Upper bound
Age	0.020	0.024	0.058	0.86	0.390	−0.026	0.067
Education	−0.235	0.117	−0.136	−2.00	0.047*	−0.467	−0.003
Gravidity (the number of times that a woman has been pregnant, including current pregnancy)	−0.234	0.088	−0.182	−2.66	0.008*	−0.407	− 0.061
Chromosomal abnormality in previous pregnancy	−0.867	1.228	−0.042	− 0.71	0.481	−3.292	1.557
Screening result (normal as the referent)							
Abnormal result	−0.177	1.257	−0.009	−0.14	0.888	−2.659	2.305
Don’t know yet	0.131	0.252	0.033	0.52	0.605	−0.367	0.628
Activeness	0.072	0.031	0.176	2.30	0.023*	0.010	0.134
Informedness	−0.004	0.017	−0.022	− 0.26	0.793	− 0.037	0.029
Confidence	0.115	0.024	0.388	4.74	< 0.001**	0.067	0.164
Social pressure	−0.277	0.062	−0.306	−4.50	< 0.001**	− 0.399	−0.155
Difficulty	−0.162	0.043	−0.274	−3.75	< 0.001**	−0.248	− 0.077
Negative emotion	0.004	0.016	0.020	0.26	0.798	−0.028	0.036
Positive emotion	−0.007	0.040	−0.013	−0.16	0.871	−0.086	0.072

R^2^ = 0.44; *p<0.05; **p<0.001

## Discussion

### “Balancing” recommendation

By integrating qualitative and quantitative analysis, one of our principal observations is that when there is a population risk of aneuploidy but the risk has not been individually revealed, women’s activeness in choice-making is not very high. A document with clear information on chromosomal screening, the offered test options and the possible results of the tests may be sufficient for women to read and be informed. Some features of the different methods, including the testing coverage, timing and accuracy should be clearly presented. Another important observation is that although they were offered the booklet shown in Additional file 1, the women in our study were not well informed and many of them were not aware of the STS possibility. The possible explanations are that they regarded the firstly presented option as the best option, they relied solely on public health nurses’ advice or they thought that in their situation it was not necessary to read the information extensively. The regression result indicates that information and knowledge is not the decisive factor for choice satisfaction in prenatal screening. Gourounti et al. [43] have made a similar observation that knowledge component was not decisive for the calculation of informed choice. In general, women do not spend a lot of time considering the screening options. Making an easy and quick choice is what they appear to expect. Recommendation with backup options given by health professionals may work well in this context.

Our study indicates that the recommendation and advice from health professionals (such as the public health nurse of maternity care unit) is highly important for women to make decisions. It is in line with the finding by Aune and Möller [25]: although women knew the health professionals were neutral in their statements and provided ‘balanced’ information and non-directive counseling, there was still a request for more advice and suggestions. According to Porter and Macintyre [44], women tend to assume that whatever the service system offers is well thought and likely the best option. However, this creates a danger of viewing everything the system suggests as routine procedures that do not require a decision [26]. Therefore, even though in previous studies it has been suggested that women did not see such system recommendations as being incompatible with making autonomous choices, there is a need for clarification of the nature of the discussion and counseling. Guidelines on how to support women’s choice-making based on their capability, willingness and preferences should be clearly defined [8].

### Constructing active choice environment

A large body of research indicates that pregnant women want to be part of the decision-making process in prenatal screening and testing and make autonomous choices [25, 45, 46]. In our study, women’s activeness in choice-making highly influences choice satisfaction. Increased active choice-making leads to higher choice satisfaction, whereas the women in our study do not make active choices in general. This may reflect that the current choice environment in Finland for prenatal screening program does not provide women with enough opportunities or motivations to make active choices. According to women’s accounts, they appreciated the recommendation from maternity care units, but they would have wanted to know the different options. Offered with recommendation together with other options, women would be given better chances to think and make choices by themselves.

Our result of women’s preference on the screening tests is consistent with many other studies: women have a strong preference for the combination of first trimester maternal serum screening and ultrasound measurement of the nuchal translucency and fetal crown-rump length, over STS, because of the earlier estimation, higher accuracy and providing more information about the baby [47–50]. However, some women have only the option of STS due to special situations such as traveling abroad occurring at the window of the FTS. Our regression analysis showed that choice-making confidence highly influences women’s choice satisfaction and preference consistency partially boosts the confidence. In order to facilitate preference-consistent choices in prenatal screening, women should be offered with opportunities to discuss their wishes and personal situations and clarify their preferences [9].

In general, women did not feel much pressure to participate in the screening tests and most of them reported they received some support from others, mostly from public health nurses of maternity care units and husbands/partners. This empirically supports Lawson and Pierson’s [51] conclusion that the women identified their physicians and their husbands as the most important individuals from whom they sought support. We can see from the quantitative study that while social pressure decreases choice satisfaction, confidence in choice-making that embraces social support can increase choice satisfaction. This is in tune with Lawson and Pierson’s [51] argument that women who are making decisions in a socially supportive context have higher decisional competence and lower decisional conflict. Therefore, the service provider should build a socially supportive choice environment and encourage women to seek support and advice not only from healthcare professionals but also from others (e.g. husband/partner and friends).

In our study, education level negatively influences women’s choice satisfaction, which means that women with higher education level have lower choice satisfaction. It is possible that more highly educated women have higher expectations and more requirements on choice environment. As the number of women with higher education will continue to increase, improving choice environment for prenatal screening demands swift actions. In addition, gravidity (i.e. the number of times that a woman has been pregnant, including current pregnancy) also strongly affect women’s choice satisfaction: the more pregnancies, the lower the choice satisfaction. We can conceive that more experienced women have more critical views about the choices and decisions, which should be taken into account by the choice architects.

### Reliability and validity

In the qualitative study, reliability was secured by the fact that two researchers carefully planned the interview protocol together, and they consulted medical and language professionals concerning the translations of the interview questions and terms. The two researchers also analyzed the interview data first separately and then together, and a strong mutual agreement on coding and categorization was reached. In addition, using the computerized data analysis package *Atlas.ti* enhanced the reliability [52]. For the quantitative study, Cronbach’s alpha was used to assess the measurements reliability, testing how well the measurement items of one variable measure the same construct or idea [52, 53]. Table 3 includes Cronbach’s alpha of each variable and the figures are acceptable, indicating reliable measurements. In this study, deriving the measurements from the interviews established the construct validity that concerns how well the measurements reflect the investigated constructs [54]. The similarity between the findings from the interviews and the survey confirmed a certain level of construct validity [55]. Specifically, the acceptable results of confirmatory factor analysis (CFA) [56] indicate convergent and discriminant validity of this study (Additional file 7).

### Limitation and future study

One limitation in our study was the low response rate of the survey. This was probably due to asking the respondents to return the survey by mail (with prepaid envelopes) instead of collecting the survey answers at the Screening Unit. Furthermore, we did not set any deadlines for returning the questionnaires and this could lead to many respondents forgetting to return the survey. The other considered issue was that some results could be biased by the generally high education level of women in our sample, which applied to both the interviews and the survey. The generalization of the results may suffer somewhat due to this, as education level has been found to be a significant variable predictive for the choice of tests [13]. It can also be suspected that the beliefs and values of the women with different education levels differ to a certain extent [11]. However, this sample bias should be considered in the nationwide context. According to reports of Statistics Finland, the women in the district of Helsinki and Uusimaa, i.e. in the capital area, are the most highly educated women in whole of Finland [57]. This demographic composition naturally influences the likelihood of getting more answers from women with higher education. There is also a tendency of people who are academically inclined to be more willing to participate in surveys and other studies.

Despite the rather low response rate and slight education sample bias, we consider that the study sample can well represent the maternity population in the hospital district of Helsinki and Uusimaa. According to the Finnish perinatal statistics report for 2015, the mean age of our sample (32) is very close to the mean age of the parturients in Helsinki and Uusimaa region (31,4) [58]. And in terms of the family status, the study group (Married: 55.19%; Co-habitation: 38.80%) is close to the parturient population of the whole country in 2015 [58].

For further study, we plan to expand the sample to include other groups of women (e.g. women who take invasive tests, or women have a lower education status) or cover other regions or other countries. We will conduct the research in other medical fields (e.g. cancer screening) to test the generalization of our conclusions. Meanwhile, we could further the study by addressing the possible influence of personal beliefs and social values on choice-making experience and choice satisfaction.

## Conclusions

This study made contributions in the research field of women’s choice and experiences in prenatal screening and testing for common aneuploidies. Our study comprehensively investigated women’s choice-making processes from women’s perspective, explored women’s choice-making experiences and identified the experiential factors that may influence choice satisfaction in prenatal screening. In the qualitative study, we structured women’s choice-making processes and experiences in prenatal screening with six blocks: active choice (attitude and behavior), informedness (information needs and sources), preferences/considerations, social influence (perceived social support and pressure), difficulty and emotional status. Seven variables were derived from the six blocks to quantified women’s choice-making experiences, named with “activeness”, “informedness”, “confidence”, “social pressure”, “difficulty”, “positive emotion” and “negative emotion”. By regression, we found that after controlling for some demographic factors (age, education, gravidity and chromosomal abnormality in previous pregnancy) and screening results, there were four experiential factors that significantly influenced women’s choice satisfaction in prenatal screening, including activeness, confidence, perceived social pressure and perceived difficulty. Activeness in choice-making increased choice satisfaction. Choice satisfaction increased if the women had more confidence when making choices. Perceived social pressure and difficulty in choice-making negatively affected choice satisfaction.

## Additional files

Additional file 1: Neuvola booklet of the prenatal screening and testing program in Finland (English). (PDF 181 kb)

Additional file 2: Decisional Conflict Scale. (PDF 116 kb)

Additional file 3: Short-form of the State Scale of the Spielberger State-Trait Anxiety Inventory. (PDF 15 kb)

Additional file 4: Measurements (before factor analysis). (DOCX 16 kb)

Additional file 5: Factor analysis result. (DOCX 13 kb)

Additional file 6: Measurements (after factor analysis). (DOCX 17 kb)

Additional file 7: CFA result. (DOCX 18 kb)

## Abbreviations

CFA: Confirmatory factor analysis
FTS: First trimester combined screening, a screening method that takes into account the mother’s age, results of the fetal anatomy and nuchal translucency measurement by ultrasound, and results from maternal serum biomarkers (free β-human chorionic gonadotropin (β-hCG), pregnancy-associated plasma protein-A (PAPP-A)
FMC: Fetomaternal Medical Center of Helsinki University Hospital
HUS: Helsinki and Uusimaa Hospital District, Finland
Neuvola: Finnish community-based maternity care unit
STS: Second trimester serum screening, a screening method that is based on maternal serum β-hCG and alpha fetoprotein (AFP) adjusted for the gestational age; it does not involve ultrasound
TPB: Theory of Planned Behavior, A theory that links beliefs and behavior and assumes that a choice is consistent with the choice maker’s goals, expectations, and values. It analyzes and predicts individual’s choice with three constructs: behavioral beliefs, subjective norms and perceived behavioral control
