# Supplementary material for: Facilitating autonomous, confident and satisfying choices: a mixed-method study of women’s choice-making in prenatal screening for common aneuploidies
Source: BMC Pregnancy Childbirth. 2018 May 2;18:119. doi: 10.1186/s12884-018-1752-y (PMC5930782; doi:10.1186/s12884-018-1752-y)
Supplement: Supplementary file 1 — Neuvola booklet of the prenatal screening and testing program in Finland (English). (PDF 181 kb) [file 12884_2018_1752_MOESM1_ESM.pdf]

This information booklet is intended for all expectant parents. It is hoped that the parents read the booklet together. Participation in prenatal screening is voluntary.

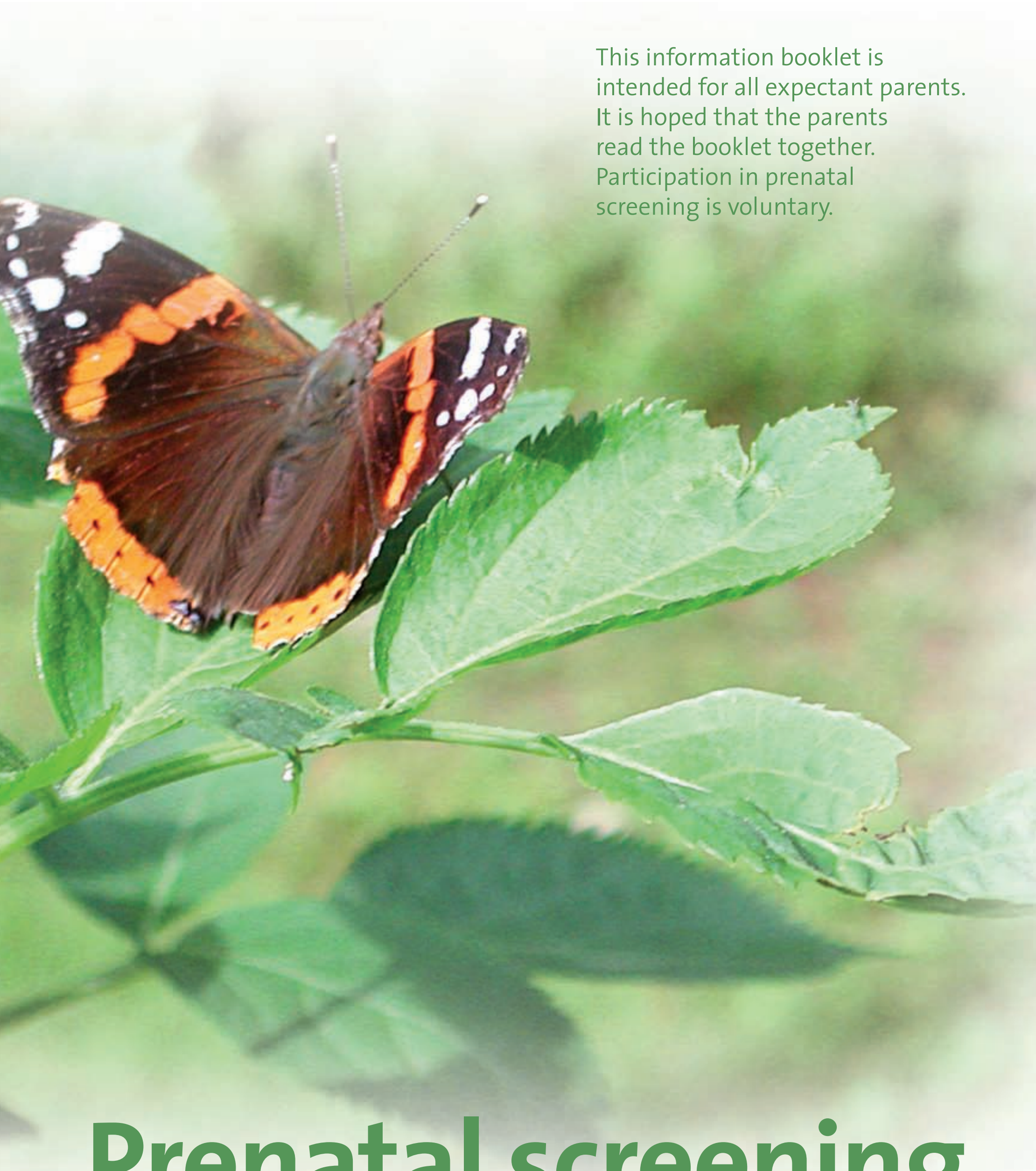

Prenatal screening

A GUIDE FOR EXPECTANT PARENTS

Information about screening tests for chromosomal and structural abnormalities

Why are there prenatal screening tests for abnormalities?

All screening tests are voluntary. It is the family and ultimately the pregnant woman herself who decide whether to participate in the tests.

A number of different tests are offered to expectant mothers in prenatal and maternity hospital clinics to find out whether the pregnancy is developing normally and to get information about possible risks.

Every expectant parent hopes for a normal development of the foetus and a healthy child. This wish is fulfilled in most cases but not always. About three in a hundred newborn children are found to have a structural or chromosomal abnormality. One in a hundred children is found to have a severe abnormality. Some abnormalities can be detected by means of tests done during pregnancy.

However, there are many foetal diseases and structural abnormalities that cannot be detected during pregnancy. The screening methods described in this booklet have been selected and scheduled so as to make sure that they are as accurate and reliable as possible.

Many expectant parents wish to be informed about possible foetal abnormalities as this may help monitor the pregnancy and prepare for delivery. Detecting a foetal disease before delivery may also help in planning the care of the newborn child. If a severe foetal disease or defect or an increased risk of a severe disease or defect is found, the expectant parents will have an opportunity to decide on possible termination of pregnancy.

Before making the decision to participate in the screening programme, the expectant parents should also consider the consequences that the decision might have. Fortunately, test results are mostly normal. However, it is possible that the results indicate an increased risk of a defect or disease. After the test results are available, you will have only a little time for consideration in order to decide on further tests and whether to continue or terminate the pregnancy.

What do the screening tests look for and what are they able to find?

The maternity clinic sees to it that the examinations are carried out at the right time.

The risk of abnormality increases with the mother's age. Some chromosomal abnormalities can be screened by tests done during pregnancy. The most common chromosomal abnormality is Down syndrome (trisomy 21). A screening test may also reveal an increased risk of other chromosomal abnormalities. Various structural abnormalities, in turn, can be detected by ultrasound examinations even if the chromosomes were found to be normal.

Early pregnancy general ultrasound examination

Early Pregnancy General Ultrasound at 10–13 weeks of pregnancy is available for all pregnant women. The main purpose of the examination is to confirm that the foetus is alive and to accurately determine the duration of pregnancy, as well as to find out how many foetuses there are. The examination is painless and harmless to the foetus. Although the purpose is not to look for foetal abnormalities, some significant and severe structural abnormalities may already be revealed in the examination. If there is a suspicion of abnormality, the expectant parents will be informed. However, many structural abnormalities cannot yet be seen at this stage of pregnancy.

Screening for foetal abnormalities

■ *A nuchal translucency (NT) scan can be combined with the early pregnancy general ultrasound. It measures the amount of fluid behind the neck of the foetus in order to screen for chromosomal abnormalities. Please tell your doctor or nurse before the general ultrasound if you want to participate in the NT screening for chromosomal abnormalities.*

EARLY PREGNANCY COMBINED SCREENING

Early Pregnancy Combined Screening is the primary method of screening chromosomal abnormalities: *A blood test is taken from the pregnant woman at 9–11 weeks of pregnancy. The general ultrasound is scheduled at 11–13 weeks of pregnancy and the NT scan is carried out at the same time*. When the results of the blood test are combined with the NT scan results, it is possible to identify a foetus at increased risk of chromosomal abnormality.

You will be informed about the results of the chromosome screening test by your own prenatal clinic or a regional prenatal screening unit.

Falling into the risk group at the first screening stage does not yet mean that the foetus would have a disease or defect.

If the combined screening test indicates an increased risk of chromosomal abnormality, the expectant parents are offered an opportunity to have a prenatal chromosome test done using a sample of the placenta or amniotic fluid. These further tests establish whether the foetus has normal chromosomes. About one in one to two hundred tests where a sample is taken of the mother's placenta or amniotic fluid lead to a miscarriage.

If the combined screening test cannot be performed, a second trimester blood screening test can be done at 15–16 weeks of pregnancy.

No screening methods where a sample is taken of the mother's blood can be used in twin or other multiple

pregnancies. However, the risk of chromosomal abnormality can be assessed even in this case by measuring the amount of fluid behind the back of the foetus using the NT scan.

The older the mother is, the higher the risk of chromosomal abnormalities. If the mother is aged 40 or over, she may be offered a prenatal chromosome test done directly from a placenta or amniotic fluid sample without preceding screening.

STRUCTURAL ULTRASOUND EXAMINATION

Structural Ultrasound at 18–21 weeks of pregnancy is available for all pregnant women. It is a systematic examination of foetal organs. The examination is painless and harmless to the foetus.

The results are reported to the mother at the same time as the ultrasound examination is done. An abnormal result is always confirmed by a new examination, which is often performed in another hospital. However, the necessary further testing is planned as required according to each detected abnormality.

ULTRASOUND EXAMINATION AFTER 24+0 WEEKS OF PREGNANCY

From 24+0 weeks of pregnancy onwards, a termination of pregnancy on the grounds of a foetal abnormality is no longer possible. Nevertheless, the detection of structural abnormalities may help plan the care of the child to be born. This ultrasound examination can be performed in place of the examination to be performed at 18–21 weeks of pregnancy.

The screening programmes offered to families comply with the Government Decrees on Screening Programmes No. 1339/2006 and 280/2009. For further information in Finnish or Swedish please visit: www.stm.fi

Simplified representation of the optio

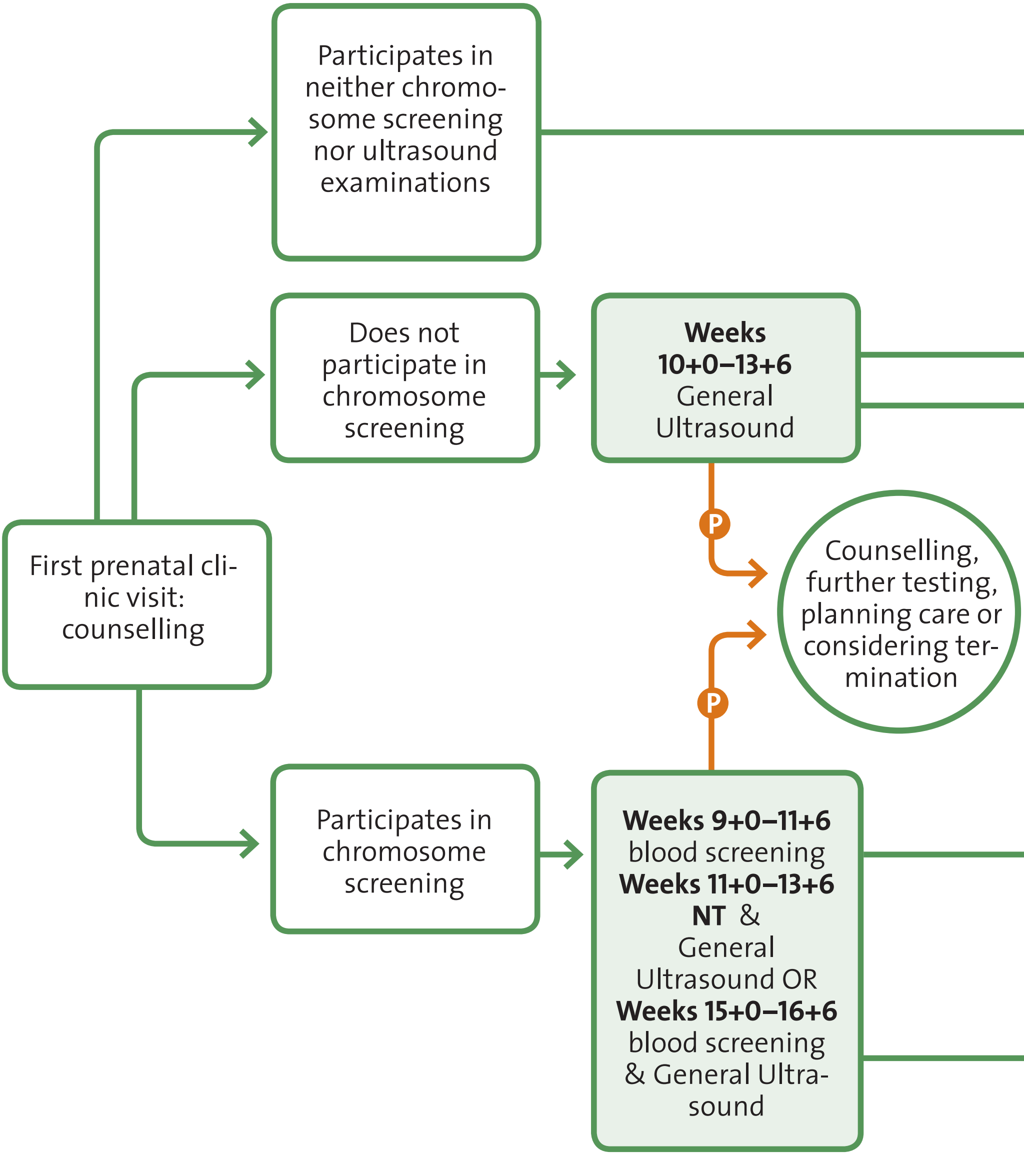

ns available in prenatal screening

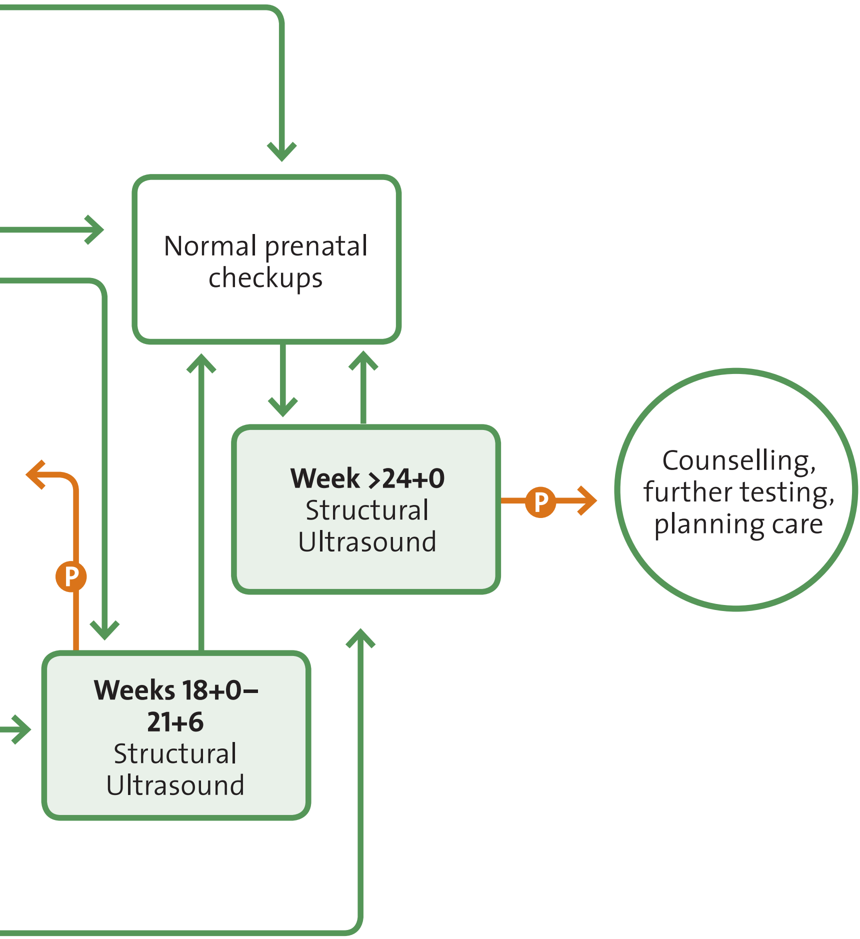

P =abnormal result; **NT** = nuchal translucency;
Weeks =weeks of pregnancy. The duration of pregnancy is expressed as weeks + days.

The text has been written by Docent Jaana Leipälä, Professor Jaakko Ignatius, Docent Ilona Autti-Rämö and Professor Marjukka Mäkelä. *© Authors and THL* *• Layout: Harri Heikkilä • Cover photo: Marjukka Mäkelä • Helsinki University Print, Helsinki, Finland 2009*

NATIONAL INSTITUTE
FOR HEALTH AND WELFARE

Finohta

KID009
ISBN 978-952-245-158-3 (PRINTED)
ISBN 978-952-245-159-0 (PDF)

PUBLICATION SALES
www.thl.fi/bookshop
Phone: +358 20 610 7190
Fax: +358 20 610 7450
