# Supplementary material for: Facilitating autonomous, confident and satisfying choices: a mixed-method study of women’s choice-making in prenatal screening for common aneuploidies
Source: BMC Pregnancy Childbirth. 2018 May 2;18:119. doi: 10.1186/s12884-018-1752-y (PMC5930782; doi:10.1186/s12884-018-1752-y)
Supplement: Supplementary file 2 — Decisional Conflict Scale. (PDF 116 kb) [file 12884_2018_1752_MOESM2_ESM.pdf]

Additional file 2. Decisional Conflict Scale

	Strongly Agree [0]	Agree [1]	Neither Agree Or Disagree [2]	Disagree [3]	Strongly Disagree [4]
1. I know which options are available to me.	☐	☐	☐	☐	☐
2. I know the benefits of each option.	☐	☐	☐	☐	☐
3. I know the risks and side effects of each option.	☐	☐	☐	☐	☐
4. I am clear about which benefits matter most to me.	☐	☐	☐	☐	☐
5. I am clear about which risks and side effects matter most.	☐	☐	☐	☐	☐
6. I am clear about which is more important to me (the benefits or the risks and side effects).	☐	☐	☐	☐	☐
7. I have enough support from others to make a choice.	☐	☐	☐	☐	☐
8. I am choosing without pressure from others.	☐	☐	☐	☐	☐
9. I have enough advice to make a choice.	☐	☐	☐	☐	☐
10. I am clear about the best choice for me.	☐	☐	☐	☐	☐
11. I feel sure about what to choose.	☐	☐	☐	☐	☐
12. This decision is easy for me to make.	☐	☐	☐	☐	☐
13. I feel I have made an informed choice.	☐	☐	☐	☐	☐
14. My decision shows what is important to me.	☐	☐	☐	☐	☐
15. I expect to stick with my decision.	☐	☐	☐	☐	☐
16. I am satisfied with my decision.	☐	☐	☐	☐	☐

Source: O'Connor AM. User Manual-Decisional Conflict Scale. 1993 [updated 2010]. In. Ottawa: Ottawa Hospital Research Institute 2010.
