# Supplementary material for: Facilitating autonomous, confident and satisfying choices: a mixed-method study of women’s choice-making in prenatal screening for common aneuploidies
Source: BMC Pregnancy Childbirth. 2018 May 2;18:119. doi: 10.1186/s12884-018-1752-y (PMC5930782; doi:10.1186/s12884-018-1752-y)
Supplement: Supplementary file 3 — Short-form of the State Scale of the Spielberger State-Trait Anxiety Inventory. (PDF 15 kb) [file 12884_2018_1752_MOESM3_ESM.pdf]

Additional file 3. Short-form of the State Scale of the Spielberger State-Trait Anxiety Inventory

	Not at all	Somewhat	Moderately	Very much
1. I feel calm	1	2	3	4
2. I am tense	1	2	3	4
3. I feel upset	1	2	3	4
4. I am relaxed	1	2	3	4
5. I feel content	1	2	3	4
6. I am worried	1	2	3	4

Source: Marteau TM, Bekker H: The development of a six-item short-form of the state scale of the Spielberger State-Trait Anxiety Inventory (STAI). Br J Clin Psychol 1992, 31(3):301-306.
