# Supplementary material for: Facilitating autonomous, confident and satisfying choices: a mixed-method study of women’s choice-making in prenatal screening for common aneuploidies
Source: BMC Pregnancy Childbirth. 2018 May 2;18:119. doi: 10.1186/s12884-018-1752-y (PMC5930782; doi:10.1186/s12884-018-1752-y)
Supplement: Supplementary file 5 — Factor analysis result. (DOCX 13 kb) [file 12884_2018_1752_MOESM5_ESM.docx]

**Additional file 5. Result of factor analysis for the measurement items of choice-making experience**

Rotated factor loadings (pattern matrix) and unique variances

---------------------------------------------------------------------------------------------------

Variable | Factor1 Factor2 Factor3 Factor4 Factor5 Factor6 Factor7 | Uniqueness

-------------+----------------------------------------------------------------------+--------------

activeness~d | 0.7740 | 0.3484

activeness~h | 0.6580 | 0.4588

activenes~re | 0.5834 | 0.3707

activenes~se | -0.6947 | 0.5444

activeness~t | 0.5535 | 0.6601

knowOption | 0.5407 | 0.4597

knowTheory | 0.8515 | 0.2285

knowProced~e | 0.9231 | 0.1501

knowFeature | 0.8262 | 0.2487

knowAdDisad | 0.8494 | 0.1922

knowResult | 0.8623 | 0.2449

knowNothing | -0.7183 | 0.3293

clearBenefit | 0.8315 | 0.2331

clearRisk | 0.7592 | 0.2509

showImport~e | 0.7105 | 0.3810

choicePres~e | 0.5798 | 0.4533

enoughSupp~t | 0.5892 | 0.5330

enoughAdvice | 0.5177 | 0.3502

feelAwful | 0.8141 | 0.3555

feelThinking | 0.7885 | 0.3389

feelUncont~l | 0.7961 | 0.4139

feelUncert~n | 0.7298 | 0.3347

feelPanic | 0.7562 | 0.3744

feelRestless | 0.7973 | 0.2877

feelWorried | 0.7674 | 0.2374

feelTense | 0.7290 | 0.3496

feelUpset | 0.5746 | 0.5214

feelCalm | 0.6356 | 0.3844

feelRelax | 0.7578 | 0.2293

feelContent | 0.6584 | 0.3436

difficultT~e | 0.6568 | 0.5225

lotEffort | 0.8671 | 0.1532

muchTime | 0.8327 | 0.1708

---------------------------------------------------------------------------------------------------

(blanks represent abs(loading)<.4)

Factor rotation matrix

-----------------------------------------------------------------------------

| Factor1 Factor2 Factor3 Factor4 Factor5 Factor6 Factor7

-------------+---------------------------------------------------------------

Factor1 | 0.5076 -0.6916 0.5143 0.1223 -0.3208 0.5478 0.4891

Factor2 | 0.7546 0.6479 0.5695 0.7027 0.5331 -0.2778 0.2745

Factor3 | -0.2446 -0.1603 0.0981 0.4088 0.6298 0.1976 -0.1697

Factor4 | 0.1321 0.1411 -0.2123 0.2304 -0.0813 0.5738 -0.5060

Factor5 | -0.3039 0.2015 0.5326 0.1197 -0.3141 0.3222 0.0658

Factor6 | -0.0071 0.1251 -0.1731 -0.2360 0.1900 0.3724 0.4764

Factor7 | 0.0574 0.0090 0.2068 -0.4484 0.2736 0.1098 -0.4116

-----------------------------------------------------------------------------
