# Supplementary material for: Facilitating autonomous, confident and satisfying choices: a mixed-method study of women’s choice-making in prenatal screening for common aneuploidies
Source: BMC Pregnancy Childbirth. 2018 May 2;18:119. doi: 10.1186/s12884-018-1752-y (PMC5930782; doi:10.1186/s12884-018-1752-y)
Supplement: Supplementary file 6 — Measurements (after factor analysis). (DOCX 17 kb) [file 12884_2018_1752_MOESM6_ESM.docx]

**Additional file 6. Summary of the measurements (after factor analysis)**

| **Variables** | **What to measure** | **Measurement design evidence** | **Items (statements with 5-point Likert scale )** | **Score range** |
| --- | --- | --- | --- | --- |
| Activeness | The extent to which women made active choice | Qualitative study results; Group discussion; factor analysis | - I read all relevant information about the different options. - I sought for relevant information from different sources. - I actively consulted and discussed with significant ones about the options. - I spend much time in comparing different options and thinking about possible outcomes. | 4-20 |
| Informedness | From women’s perspective, how much they know about the screening options | Informed subscale of Decisional Conflict Scale designed by O'Connor; Qualitative study results; Group discussion; factor analysis | - I knew that there were different screening programs offered to   Finnish pregnant women.   - I knew the mechanisms and theories of different programs. - I knew the procedures of different programs. - I knew features (e.g. timing, accuracy and risk) of different programs. - I knew the advantages and disadvantages of different programs. - I knew the possible results produced by different programs. - I knew nothing about the different programs. | 7-35 |
| Confidence | From women’s perspective, whether their preferences are clear, whether the choice is consistent with their preferences, and whether they got enough support from others when making choices | Some items of Informed subscale and Effective Decision subscale of Decisional Conflict Scale designed by O'Connor; Support subscale Decisional Conflict Scale designed by O'Connor; Group discussion; factor analysis | - I felt clear about which benefits matter most to me. - I felt clear about which risks and side effects matter most to me. - My choice showed what was important to me. - I had enough support from others to make the decision and choice. - I had enough advice to make the decision and choice. | 5-25 |
| Social pressure | To assess the extent of social pressure perceived by women in choice-making. | Support subscale Decisional Conflict Scale designed by O'Connor; Qualitative study results; Group discussion; factor analysis | - I was deciding and choosing without pressure from others. - Someone else (e.g. baby's father, family members, medical staff, etc.) made the choices for me. | 2-10 |
| Difficulty | From women’s perspective, whether it is difficult to make choices for screening. | Qualitative study results; Group discussion; factor analysis | - It was difficult to make decisions and choices for the screening program. - I made many efforts to think about the issues and make the choice for the screening program. - I spent much time in thinking about the issues and making the choice for the screening program. | 3-15 |
| Negative emotion | To measure women’s anxiety level when making choices for screening | Qualitative study results; Group discussion; Six-item short-form of the state scale of the Spielberger State-Trait Anxiety Inventory; factor analysis | - I felt something awful was about to happen. - I kept thinking about the possible bad consequences or results. - I felt I could not control the situation. - I felt uncertain. - I felt panic. - I felt restless - I was worried - I was tense - I felt upset | 9-45 |
| Positive emotion | To measure women’s positive emotional level when making choices for screening | Qualitative study results; Group discussion; Six-item short-form of the state scale of the Spielberger State-Trait Anxiety Inventory; factor analysis | - I felt calm - I was relaxed - I felt content | 3-15 |
| Choice satisfaction | Form women’s perspective, whether they felt satisfied with the choices. | Some items of Effective Decision subscale of Decisional Conflict Scale designed by O'Connor; Group discussion | - I expected to stick with my choice and decision. - I was satisfied with my decision and choice. | 2-10 |
