# Supplementary material for: Facilitating autonomous, confident and satisfying choices: a mixed-method study of women’s choice-making in prenatal screening for common aneuploidies
Source: BMC Pregnancy Childbirth. 2018 May 2;18:119. doi: 10.1186/s12884-018-1752-y (PMC5930782; doi:10.1186/s12884-018-1752-y)
Supplement: Supplementary file 7 — CFA result. (DOCX 18 kb) [file 12884_2018_1752_MOESM7_ESM.docx]

**Additional file 7. Result of confirmatory factor analysis for the measurements in the regression model**

-----------------------------------------------------------------------------------------------------

| OIM

| Coef. Std. Err. z P>|z| [95% Conf. Interval]

------------------------------------+----------------------------------------------------------------

Measurement |

activenessRead <- |

Activeness | 1 (constrained)

_cons | 2.594444 .0954836 27.17 0.000 2.4073 2.781589

----------------------------------+----------------------------------------------------------------

activenessSearch <- |

Activeness | .9637533 .0934124 10.32 0.000 .7806683 1.146838

_cons | 2.433333 .1012575 24.03 0.000 2.234872 2.631794

----------------------------------+----------------------------------------------------------------

activenessCompare <- |

Activeness | .7754405 .0788696 9.83 0.000 .6208589 .9300222

_cons | 1.911111 .0770958 24.79 0.000 1.760006 2.062216

----------------------------------+----------------------------------------------------------------

activenessConsult <- |

Activeness | .5239342 .0799579 6.55 0.000 .3672195 .6806488

_cons | 1.772222 .0792833 22.35 0.000 1.61683 1.927615

----------------------------------+----------------------------------------------------------------

knowOption <- |

Informedness | 1 (constrained)

_cons | 4.05 .0920531 44.00 0.000 3.869579 4.230421

----------------------------------+----------------------------------------------------------------

knowTheory <- |

Informedness | 1.335771 .1290588 10.35 0.000 1.08282 1.588722

_cons | 2.75 .092588 29.70 0.000 2.568531 2.931469

----------------------------------+----------------------------------------------------------------

knowProcedure <- |

Informedness | 1.420985 .1318352 10.78 0.000 1.162592 1.679377

_cons | 2.883333 .0933846 30.88 0.000 2.700303 3.066364

----------------------------------+----------------------------------------------------------------

knowFeature <- |

Informedness | 1.353373 .1319173 10.26 0.000 1.09482 1.611926

_cons | 2.933333 .0941499 31.16 0.000 2.748803 3.117864

----------------------------------+----------------------------------------------------------------

knowAdDisad <- |

Informedness | 1.311802 .1268524 10.34 0.000 1.063176 1.560428

_cons | 2.911111 .0900236 32.34 0.000 2.734668 3.087554

----------------------------------+----------------------------------------------------------------

knowResult <- |

Informedness | 1.217987 .122425 9.95 0.000 .9780383 1.457936

_cons | 3.277778 .0882111 37.16 0.000 3.104887 3.450668

----------------------------------+----------------------------------------------------------------

knowNothing <- |

Informedness | -1.27317 .1420612 -8.96 0.000 -1.551604 -.9947349

_cons | 2.105556 .1052619 20.00 0.000 1.899246 2.311865

----------------------------------+----------------------------------------------------------------

enoughSupport <- |

ChoiceConf | 1 (constrained)

_cons | 3.455556 .0930691 37.13 0.000 3.273143 3.637968

----------------------------------+----------------------------------------------------------------

enoughAdvice <- |

ChoiceConf | 1.260393 .1576657 7.99 0.000 .9513738 1.569412

_cons | 3.5 .0954198 36.68 0.000 3.312981 3.687019

----------------------------------+----------------------------------------------------------------

clearBenefit <- |

ChoiceConf | 1.496851 .1795244 8.34 0.000 1.144989 1.848712

_cons | 3.305556 .0956128 34.57 0.000 3.118158 3.492953

----------------------------------+----------------------------------------------------------------

clearRisk <- |

ChoiceConf | 1.44016 .1756833 8.20 0.000 1.095826 1.784493

_cons | 3.105556 .0947698 32.77 0.000 2.91981 3.291301

----------------------------------+----------------------------------------------------------------

showImportance <- |

ChoiceConf | 1.209848 .1532122 7.90 0.000 .9095572 1.510138

_cons | 3.527778 .092773 38.03 0.000 3.345946 3.709609

----------------------------------+----------------------------------------------------------------

choicePressure <- |

SocialPress | 1 (constrained)

_cons | 4.394444 .080698 54.46 0.000 4.236279 4.55261

----------------------------------+----------------------------------------------------------------

activenessSomeoneelse <- |

SocialPress | -.434429 .0909072 -4.78 0.000 -.6126038 -.2562542

_cons | 1.322222 .0650999 20.31 0.000 1.194629 1.449816

----------------------------------+----------------------------------------------------------------

difficultToChoose <- |

Difficulty | 1 (constrained)

_cons | 1.888889 .0793061 23.82 0.000 1.733452 2.044326

----------------------------------+----------------------------------------------------------------

lotEffort <- |

Difficulty | 1.498207 .1705351 8.79 0.000 1.163964 1.832449

_cons | 1.833333 .0713624 25.69 0.000 1.693466 1.973201

----------------------------------+----------------------------------------------------------------

muchTime <- |

Difficulty | 1.451562 .1706891 8.50 0.000 1.117018 1.786107

_cons | 1.783333 .0708306 25.18 0.000 1.644508 1.922159

----------------------------------+----------------------------------------------------------------

feelAwful <- |

NegaFeel | 1 (constrained)

_cons | 1.644444 .0669541 24.56 0.000 1.513217 1.775672

----------------------------------+----------------------------------------------------------------

feelThinking <- |

NegaFeel | 1.345251 .1252009 10.74 0.000 1.099862 1.59064

_cons | 2.3 .0827386 27.80 0.000 2.137835 2.462165

----------------------------------+----------------------------------------------------------------

feelUncontrol <- |

NegaFeel | 1.113608 .1164696 9.56 0.000 .8853322 1.341885

_cons | 1.722222 .0757903 22.72 0.000 1.573676 1.870768

----------------------------------+----------------------------------------------------------------

feelUncertain <- |

NegaFeel | 1.606032 .1474394 10.89 0.000 1.317056 1.895008

_cons | 2.361111 .0955051 24.72 0.000 2.173925 2.548298

----------------------------------+----------------------------------------------------------------

feelPanic <- |

NegaFeel | .8513115 .0870164 9.78 0.000 .6807625 1.021861

_cons | 1.433333 .0567102 25.27 0.000 1.322183 1.544483

----------------------------------+----------------------------------------------------------------

feelRestless <- |

NegaFeel | 1.511807 .133733 11.30 0.000 1.249695 1.773919

_cons | 2.111111 .0870961 24.24 0.000 1.940406 2.281816

----------------------------------+----------------------------------------------------------------

feelWorried <- |

NegaFeel | 1.649127 .1440432 11.45 0.000 1.366807 1.931447

_cons | 2.555556 .0940259 27.18 0.000 2.371268 2.739843

----------------------------------+----------------------------------------------------------------

feelTense <- |

NegaFeel | 1.45852 .1358698 10.73 0.000 1.19222 1.724819

_cons | 2.111111 .087802 24.04 0.000 1.939022 2.2832

----------------------------------+----------------------------------------------------------------

feelUpset <- |

NegaFeel | .6813162 .0899958 7.57 0.000 .5049277 .8577047

_cons | 1.383333 .0584126 23.68 0.000 1.268847 1.49782

----------------------------------+----------------------------------------------------------------

feelCalm <- |

PosiFeel | 1 (constrained)

_cons | 3.405556 .0826629 41.20 0.000 3.243539 3.567572

----------------------------------+----------------------------------------------------------------

feelRelax <- |

PosiFeel | 1.22604 .0973592 12.59 0.000 1.035219 1.41686

_cons | 3.127778 .0874998 35.75 0.000 2.956281 3.299274

----------------------------------+----------------------------------------------------------------

feelContent <- |

PosiFeel | 1.048277 .0926496 11.31 0.000 .8666874 1.229867

_cons | 3.4 .0829993 40.96 0.000 3.237324 3.562676

----------------------------------+----------------------------------------------------------------

satisfiedChoice <- |

ChoiceSatisfaction | 1 (constrained)

_cons | 4.6 .0558879 82.31 0.000 4.490462 4.709538

----------------------------------+----------------------------------------------------------------

stickToChoice <- |

ChoiceSatisfaction | 1.75692 .2584056 6.80 0.000 1.250455 2.263386

_cons | 4.25 .0765277 55.54 0.000 4.100009 4.399991

------------------------------------+----------------------------------------------------------------

var(e.activenessRead)| .5644084 .0950843 .4056897 .785223

var(e.activenessSearch)| .8455209 .1140517 .6490922 1.101393

var(e.activenessCompare)| .4224652 .0642228 .3136115 .5691018

var(e.activenessConsult)| .8358967 .0937893 .670882 1.0415

var(e.knowOption)| .8607579 .0943756 .6943097 1.067109

var(e.knowTheory)| .3573636 .0463146 .2772004 .4607089

var(e.knowProcedure)| .2279257 .0355552 .1678844 .30944

var(e.knowFeature)| .3784083 .0473143 .2961629 .4834935

var(e.knowAdDisad)| .3152432 .0415757 .2434363 .4082311

var(e.knowResult)| .4148073 .0509515 .3260557 .5277168

var(e.knowNothing)| .9172529 .1031729 .735776 1.14349

var(e.enoughSupport)| .9960013 .113612 .7964617 1.245532

var(e.enoughAdvice)| .7442989 .0960599 .5779503 .9585266

var(e.clearBenefit)| .3837872 .0680994 .2710522 .5434105

var(e.clearRisk)| .4486615 .0715342 .3282494 .6132445

var(e.showImportance)| .7249509 .0889683 .5699627 .9220847

var(e.choicePressure)| .0995474 .1839719 .0026603 3.724964

var(e.activenessSomeoneelse)| .560401 .0684912 .4410284 .712084

var(e.difficultToChoose)| .7628267 .0830225 .6162899 .9442057

var(e.lotEffort)| .0877792 .0324241 .0425572 .181055

var(e.muchTime)| .1249772 .0320947 .0755507 .2067392

var(e.feelAwful)| .3789382 .0434798 .302622 .4745

var(e.feelThinking)| .4577153 .0547818 .3620088 .5787243

var(e.feelUncontrol)| .5032084 .0572063 .4026991 .6288037

var(e.feelUncertain)| .5379278 .0659428 .4230367 .6840218

var(e.feelPanic)| .2687218 .0308967 .2145037 .3366442

var(e.feelRestless)| .3872686 .0491218 .3020261 .4965695

var(e.feelWorried)| .4274282 .0557591 .3309952 .5519562

var(e.feelTense)| .4772315 .0578052 .3763798 .6051066

var(e.feelUpset)| .4155041 .0454681 .3352967 .5148981

var(e.feelCalm)| .4812338 .0629854 .3723474 .6219623

var(e.feelRelax)| .2526379 .0576392 .1615464 .3950933

var(e.feelContent)| .4172258 .0589318 .316331 .5503013

var(e.satisfiedChoice)| .3503616 .0449696 .2724351 .4505779

var(e.stickToChoice)| .4002019 .0897348 .2578811 .6210674

var(Activeness)| 1.076672 .178566 .7778782 1.490236

var(Informedness)| .6645198 .1352475 .445929 .9902621

var(ChoiceConf)| .5631343 .1336894 .3536184 .8967868

var(SocialPress)| 1.072644 .2211164 .716123 1.606658

var(Difficulty)| .3692765 .0905649 .2283467 .5971844

var(NegaFeel)| .4279752 .0770329 .3007514 .6090172

var(PosiFeel)| .7487353 .1250198 .5397581 1.038622

var(ChoiceSatisfaction)| .2118606 .052977 .1297783 .3458584

------------------------------------+----------------------------------------------------------------

cov(Activeness,Informedness)| .4569678 .0907808 5.03 0.000 .2790408 .6348949

cov(Activeness,ChoiceConf)| .4072109 .0879858 4.63 0.000 .2347619 .5796598

cov(Activeness,SocialPress)| .2755919 .0956469 2.88 0.004 .0881274 .4630563

cov(Activeness,Difficulty)| .3823304 .0744885 5.13 0.000 .2363356 .5283252

cov(Activeness,NegaFeel)| .2338226 .0627923 3.72 0.000 .1107519 .3568933

cov(Activeness,PosiFeel)| -.1362919 .0796009 -1.71 0.087 -.2923068 .019723

cov(Activeness,ChoiceSatisfaction)| .1790209 .0547109 3.27 0.001 .0717895 .2862524

cov(Informedness,ChoiceConf)| .4033895 .0791166 5.10 0.000 .2483239 .5584551

cov(Informedness,SocialPress)| .3151253 .076685 4.11 0.000 .1648255 .4654251

cov(Informedness,Difficulty)| .1452182 .0449559 3.23 0.001 .0571063 .2333301

cov(Informedness,NegaFeel)| .0768269 .0434025 1.77 0.077 -.0082403 .1618942

cov(Informedness,PosiFeel)| .0017338 .0572528 0.03 0.976 -.1104795 .1139472

cov(Informedness,ChoiceSatisfaction)| .139904 .0400211 3.50 0.000 .0614641 .2183438

cov(ChoiceConf,SocialPress)| .4300678 .0868288 4.95 0.000 .2598865 .6002492

cov(ChoiceConf,Difficulty)| .1245304 .0420439 2.96 0.003 .0421258 .206935

cov(ChoiceConf,NegaFeel)| .0397472 .040358 0.98 0.325 -.0393529 .1188474

cov(ChoiceConf,PosiFeel)| .0739902 .0555854 1.33 0.183 -.0349552 .1829355

cov(ChoiceConf,ChoiceSatisfaction)| .2323933 .0506176 4.59 0.000 .1331846 .331602

cov(SocialPress,Difficulty)| .0698035 .0517483 1.35 0.177 -.0316213 .1712282

cov(SocialPress,NegaFeel)| -.0465573 .0548153 -0.85 0.396 -.1539933 .0608788

cov(SocialPress,PosiFeel)| .168846 .0761998 2.22 0.027 .0194971 .3181949

cov(SocialPress,ChoiceSatisfaction)| .3188176 .061332 5.20 0.000 .1986091 .4390261

cov(Difficulty,NegaFeel)| .1709728 .0408336 4.19 0.000 .0909405 .2510052

cov(Difficulty,PosiFeel)| -.1948169 .0517446 -3.76 0.000 -.2962343 -.0933994

cov(Difficulty,ChoiceSatisfaction)| -.0012776 .0258781 -0.05 0.961 -.0519978 .0494425

cov(NegaFeel,PosiFeel)| -.3739111 .0642384 -5.82 0.000 -.4998162 -.2480061

cov(NegaFeel,ChoiceSatisfaction)| -.0097009 .028224 -0.34 0.731 -.0650189 .0456171

cov(PosiFeel,ChoiceSatisfaction)| .0677577 .0398914 1.70 0.089 -.0104279 .1459434

-----------------------------------------------------------------------------------------------------
